# Supplemental Impact of Marine Red Seaweed (*Halymenia palmata*) on the Growth Performance, Total Tract Nutrient Digestibility, Blood Profiles, Intestine Histomorphology, Meat Quality, Fecal Gas Emission, and Microbial Counts in Broilers

**DOI:** 10.3390/ani11051244

**Published:** 2021-04-27

**Authors:** Balamuralikrishnan Balasubramanian, Sureshkumar Shanmugam, Sungkwon Park, Neeraja Recharla, Jin Su Koo, Ines Andretta, In Ho Kim

**Affiliations:** 1 Department of Food Science and Biotechnology, College of Life Science, Sejong University, Seoul 05006, Korea; sungkwonpark@sejong.ac.kr (S.P.); neeruphysio39@gmail.com (N.R.); gkjs7303@gmail.com (J.S.K.); 2 Department of Animal Resource and Science, Dankook University, Cheonan 31116, Korea; sureshbiogenetic@gmail.com; 3 Department of Animal Science, Faculdade de Agronomia, Universidade Federal do Rio Grande do Sul, Porto Alegre, Rio Grande do Sul 91540-000, Brazil; ines.andretta@ufrgs.br

**Keywords:** antibiotic alternative, feed additive, feeding, poultry

## Abstract

**Simple Summary:**

Seaweed has potential bioactive substances and essential nutrients, especially polysaccharides and trace elements. Further, marine seaweeds have prebiotic effects to enhance the performance in animals and is a potential antibiotic replacer. However, neither *Halymenia palmata* nor other non-calcareous red algae have received much attention as an animal feed in modern scientific literature. Therefore, for this study, we used marine red seaweed, otherwise known as *Palmaria palmata* which, to the best of our knowledge, is the first time *H. palmata* has been evaluated as a poultry feed additive. Additionally, this dietary seaweed supplement showed beneficial effects on growth performance, relative organ weight in broilers, nutrient digestibility, fecal microbial counts, and gut health. Hence, this study suggests that a dietary seaweed supplement for broilers could be a potential option for a feed additive in the livestock sector.

**Abstract:**

The present study was conducted to evaluate the dietary effects of a marine red seaweed, *Palmaria palmata*, on the growth performance, blood profile, nutrient digestibility, meat quality, fecal gas emission, microbial population, and intestinal morphology of broilers. A total of 720 Ross 308 broiler chicks (1 day old), with an average body weight of 45 ± 0.50 g, were assigned to one of five dietary treatments (randomized complete block design) in a 42-day feeding trial. The five dietary treatments consisted of a basal diet (0% supplementation; control), and diets supplemented with 0.05%, 0.01%, 0.15%, or 0.25% red seaweed. Eight replicates were prepared per treatment, with each replicate consisting of 18 chicks in a cage. The results showed that there tended to be a greater increase in body weight in the seaweed-supplemented groups from day (d) 14 to 28 (*p* = 0.087) and d 28 to 42 (*p* = 0.082) compared to the control group, regardless of feed intake. Feed intake in the seaweed-supplemented groups increased linearly from d 14 to 28. A linear relationship between seaweed supplementation and the feed conversion ratio was observed from d 14 to 28 and throughout the whole experiment. The dietary inclusion of seaweed was linearly related to levels of albumin, creatinine, uric acid, and white blood cells in the broilers. Additionally, the total tract digestibility of dry matter increased linearly with an increase in seaweed supplementation. The dietary inclusion of seaweed had a beneficial effect on fecal microbes as *Lactobacillus* sp. counts increased and *Escherichia coli* and *Salmonella* sp. counts decreased on day 42. Histopathological examination of the intestine confirmed that seaweed dietary supplementation enhanced the heights and widths of the villi. Furthermore, the emission of fecal gases (NH_3_ and H_2_S) decreased linearly in broilers fed seaweed-supplemented diets. Dietary supplementation with seaweed led to improvements in meat quality traits, such as reductions in drip loss, water holding capacity, and cooking loss, as well as increases in relative organ weights. Based on these positive effects, dietary supplementation with seaweed in broilers can be considered a dietary option in poultry production.

## 1. Introduction

Poultry producers have been preferentially using cost-effective antibiotics in large quantities since 1951 [1] to improve growth performance and prevent diseases. However, the development of microbial resistance to these antibiotics causes health issues in the food chain from animals to humans. Consequently, the European Union has banned the use of antibiotic growth promoters in poultry diets since 2006 [2], thus motivating researchers to seek effective alternatives such as probiotics, prebiotics, herbal products, marine natural products, and organic acids [3]. One such potential natural alternative is seaweed, from which natural marine products that contain various biologically active components can be derived [4], as well as useful ingredients with abundant health benefits [5]. Since the 20th century, the consumption of seaweed (marine macroalgae) products has been increasing among people seeking to improve their health and diets. Holdt and Kraan [6] reported in 2011 that seaweeds contain various types of soluble fiber such as agar, carrageenan, and alginate, which are not present in terrestrial plants. Moreover, seaweeds are rich in nutrients, contain ample proteins, lipids, polyphenols, and polysaccharides, and possess antiviral and antifungal properties. Particularly, bioactive substances have been found in red seaweeds [7].

Seaweeds are considered a prime feed constituent in Norway, after the processing of seaweed-based meal from kelp started in the 1960s [8]. Seaweeds are preferentially used in livestock feed, as they contain nutrients such as chelating micro-minerals at higher concentrations than can be found in typical inorganic feed components. Additionally, they are rich in prebiotic complex carbohydrates, pigments, and polyunsaturated fatty acids, which can improve the health of consumers [9]. Red seaweeds are especially abundant in dietary fiber, vitamins, minerals, carotenoids, phlorotannins, amino acids, and other fitness-enhancing constituents and, thus, contribute raw materials to the pharmaceutical and nutraceutical industries [6]. The polysaccharides in seaweed make it an ideal prebiotic due to their potential for improving the gut microbiota [10]. The marine red seaweed *Palmaria palmata* (L.), otherwise known as red dulse (formerly known as *Halymenia palmata*), has smooth leathery fronds and is deep red in autumn and greenish/yellow in summer. Individuals can grow up to 30–40 cm in length. This species occurs in the North Atlantic and is found in moderately exposed to exposed shores in areas subject to tidal currents. Furthermore, as *P. palmata* is rich in protein, it was harvested for food and given as feed to livestock such as sheep and goats in Gotland (Sweden) and cows in Brittany (France) during the 19th and early 20th centuries [11].

Regarding poultry production, seaweed may be able to improve the immune status of birds and reduce pathogenic microbes in the digestive tract [12]. Previous studies have reported promising results in terms of animal growth and health with the use of seaweed dietary supplements [13,14]. Moreover, Dierick et al. [13] suggested that a brown seaweed (*Ascophyllum nodosum*) can be a natural replacement for antibiotics. Montserrat and Goñi [15] evaluated the effects of red (nori, *Porphyra ternera*) and brown seaweeds (wakame, *Undaria pinnatifida*) and reported alterations in microbial activities in rats. According to Hoebler et al. [16], the addition of 5% brown seaweed (*Laminaria digitate*) to the diet of pigs increased propionic and butyric acid concentrations in the large intestine. Furthermore, Kulshreshtha et al. [10] showed that red seaweeds (*Chondrus crispus* and *Sarcodiotheca gaudichaudii*) greatly improved the growth performance, egg efficiency, and overall gut health of laying hens. The dietary inclusion of seaweed also was shown to improve bird health and feed efficiency by increasing the abundance of beneficial gut bacteria and strengthening the innate host immune system [17]. Each type of seaweed has its own properties, so specific research on *P. palmata* needs to be conducted [9]. Nevertheless, based on previous studies, the application of seaweed should have positive effects on livestock. To date, no experiments on seaweed supplementation in the broiler diet have been initiated. Therefore, the aims of the present study were to evaluate the effects of adding marine red seaweed (*P. palmata*) to a soybean meal (SBM) based diet on the growth performance, total tract nutrient digestibility, excreta gas emissions, meat quality, intestine histomorphology, and fecal microbial counts of broiler chickens.

## 2. Materials and Methods

The research was conducted at the poultry research unit of Dankook University, Cheonan, Republic of Korea. The research protocol was permitted by the Animal Care and Use Committee of Dankook University (DK-1-1913).

### 2.1. Experimental Design, Birds, and Husbandry

A total of 720 Ross 308 broiler chicks (one-day-old) with an average body weight (BW) of 45 ± 0.50 g (mean ± SD) were assigned (complete random blocks) to one of five dietary treatments for a 42-day (d) trial. The five dietary treatments were SBM based diets supplemented with 0%, 0.05%, 0.10%, 0.15%, and 0.25% marine red seaweed. Each treatment had 8 replications with 18 chicks per cage. Chickens were raised at a room temperature of 33 ± 1 °C for the first 3 days. Later it was gradually reduced to 24 °C and the humidity was maintained at around 60% for the rest of the experiment. Each cage was equipped with nipple-type water troughs and a feeder that allowed birds to enjoy free access to feed and water throughout the experiment. The basal diets were formulated according to the requirements of the National Research Council (NRC) [18] (Table 1). The commercially procured marine red seaweed, *H. palmata* (Organic Whole Leaf-Dulse, Vitaminsea^®^) was evaluated as a feed additive in a powder form and proximate chemical compositions of the samples are illustrated in Table 2. 

### 2.2. Sampling and Measurements 

#### 2.2.1. Growth Performances

The nutritional diet was offered to broiler chickens for 42 days. Occurring on d 1, 7, 14, 28, and 42, broilers were weighed, and the amount of feed consumption and residual in each cage was recorded daily to evaluate the feed intake (FI). Occurring on d 42, the body-weight gain (BWG), FI, and feed conversion ratio (FCR) was calculated using the collected data. 

#### 2.2.2. Nutrient Digestibility 

Chromic oxide (Cr_2_O_3_, 2 g/kg) was added to the diets as an indigestible marker to determine the digestibility of dry matter (DM), and gross energy (GE). Occurring on day 14, 28, and 42, fresh excreta samples were collected (each cage) using aluminum foil sheets and stored at −20 °C for further analysis. Then, the freeze-dried samples were placed in a hot air-drying oven for 2 days. Later, samples were taken, pulverized well, and sieved using a 1 mm screen sieve. Chromium absorption was identified using UV spectrophotometry (Shimadzu, UV-1201, Kyoto, Japan) described by Williams et al. [19]. Feed and fecal samples were taken and placed in a Parr 6400 (Parr Instrument Co., Moline, IL, USA) oxygen bomb calorimeter and the heat combustion in the sample was measured to determine GE and DM, which were determined according to the method of Association of Official Analytical Chemists (AOAC) [20].

The apparent total tract digestibility was calculated using the following formula:

Digestibility (%) = {1 − [(Nf × Cd)/(Nd × Cf)]} × 100,

where Nf = nutrient concentration in excreta (% DM), Nd = nutrient concentration in diet (% DM), Cd = chromium concentration in diet (% DM), and Cf = chromium concentration in excreta (% DM).

#### 2.2.3. Fecal Microbial Counts

Occurring on d 14, 28, and at the end of the experiment, excreta samples were collected from 20 chickens randomly selected from each treatment (each treatment with 8 replication birds), then pooled on a cage basis, placed in an ice box, and taken to the laboratory for microbial analysis. One gram of composite excreta sample was diluted with 9 mL of 1% peptone broth (Becton, Dickinson and Co., Rutherford, NJ, USA) and homogenized. Viable bacteria *Lactobacillus* sp., *Escherichia coli*, and *Salmonella* sp. counts in the excreta samples then were determined by plating serial (10-fold) dilutions (in 10 g/L peptone solution) onto *Lactobacillus* III, MacConkey agar, and *Salmonella-Shigella* agar, respectively. The *Lactobacilli* medium III agar plates were incubated at 39 °C for 2 days, and the MacConkey, and *Salmonella-Shigella* agar plates were incubated at 37 °C for 1 day. Once agar plates were removed from the incubator, the bacterial colonies (*Lactobacillus* sp., *E. coli, and Salmonella* sp.) were counted and recorded immediately. 

#### 2.2.4. Fecal Noxious Gas Emissions

Approximately 300 g of feces were placed in a plastic box (2.6-L) with a small hole in the middle and sealed with adhesive plaster for 36 h at room temperature (28 °C) for fermentation. After fermentation, the plastic boxes were punctured, and headspace air was sampled approximately 2.0 cm above the samples at a rate of 100 mL/min. The gas emission content was determined by the gas sampling pump (Gas Detector tube, Gastec Corp, Model GV-100, Kanagawa, Japan), and Gastec detector tube No. 3L and 3 La for NH_3_; No. 4LL and 4 LK for H_2_S; No. 70 and 70 L for total mercaptans. 

#### 2.2.5. Blood Parameters

Occurring at week 4 and at the end of the experiment, 5 mL blood samples were collected from the branchial vein of 20 birds/treatment using a sterilized syringe and stored K_3_EDTA (Becton Dickinson Vacutainer Systems, Franklin Lakes, NJ, USA) The collected blood samples were taken to the laboratory and stored at 4 °C. Regarding serum analysis, approximately 3 mL blood samples were centrifuged at 4000× *g* for 15 min at 4 °C, to separate the serum. The white blood cell (WBC), red blood cells (RBC), and lymphocyte counts of the whole blood samples were determined using an automatic blood analyzer (ADVIA 120; Bayer). Protein, albumin, globulin, creatinine, uric acid, triglyceride, and phosphorus in the serum samples were analyzed with an automatic biochemical analyzer (RA-1000, Bayer Corp., Tarrytown, NY, USA) using colorimetric methods.

#### 2.2.6. Meat Quality Traits

Occurring on d 42, 20 broilers per treatment were randomly selected, weighed individually, and euthanized by cervical dislocation. Breast meat, gizzard, bursa of Fabricius, liver, spleen, and abdominal fat were then removed by a skilled person. All organs were weighed to express as a percentage of body weight. Only breast meats were stored at −20 °C for further analysis. Meat quality was evaluated using a Minolta CR410 (Minolta Co, Osaka, Japan) chromameter by measuring breast muscle color parameters of lightness (L*), redness (a*), and yellowness (b*). Simultaneously, duplicate pH values for each sample were measured using a pH meter (Testo205, Testo, Germany). Approximately, 4 g of breast muscle was taken to measure drip loss according to the plastic bag method of Honikel [21]. The water-holding capacity (WHC) was done by the described methods of Kauffman et al. [22]. Meat samples were cooked at 80 °C in a water bath to reach the core temperature of the fillet to 72 °C. Cooked samples were taken, re-weighed, and noted for statistical analysis.

#### 2.2.7. Histopathology Analysis

Ten other birds from each treatment were randomly picked at the end of the experimental trial and euthanized by cervical dislocation. Intestinal tract tissue samples were collected from the ileocolic junction (ileum), mid-gut (jejunum), and duodenum region and placed into neutral buffered formalin for fixation. The intestinal segments were fixed for morphometric analysis and histochemical staining in 10% buffered formalin solutions, respectively. Histological experiment samples were performed on 5 µm sections, stained by haematoxylin and eosin, and examined using an Olympus AX70 microscope (Olympus Cooperation, Tokyo, Japan). Regarding each sample, a number of well-oriented intact crypt-villus units were chosen in triplicate for each intestinal cross-section. The classification criteria for villus were based on the appearance of the intact lamina propria. The length of the villus was determined from the tip of the villus to the villus-crypt junction, according to Wilson et al. [23].

### 2.3. Statistical Analysis

All data were subjected to statistical analyses in a randomized complete block design using general linear model procedures of SAS/STAT (Statistical Analysis System, version 9.2, SAS Institute Inc., Cary, NC, USA) with a cage as the experimental unit. Mean values and standard errors of the mean were reported. Linear and quadratic polynomial contrasts were performed to determine the effect of marine red seaweed at inclusion levels in the diet. Statistical significance was considered when the *p*-value was less than 0.05 and tendencies were considered when the *p*-value was less than 0.10. 

## 3. Results 

Broiler FI improved linearly (*p* = 0.0369) with seaweed dietary supplementation from d 14 to 28 (Table 3). The BWG tended to be greater from d 14 to 28 (*p* = 0.087) and d 28 to 42 (*p* = 0.082) in the seaweed-supplemented groups, with a linear increase (*p* = 0.0075) observed over the entire experimental period. Additionally, seaweed supplementation was linearly associated with the FCR from d 28 to 42 (*p* = 0.028) and over the entire experimental period (*p* = 0.049). Quadratic effects were not observed for any performance-related variable. The level of seaweed supplementation did not affect any of the blood-related traits after week 4. However, linear increases in albumin, creatinine, uric acid, and the WBC count due to seaweed supplementation were observed over the duration of the experiment (Table 4).

**Figure 1:**
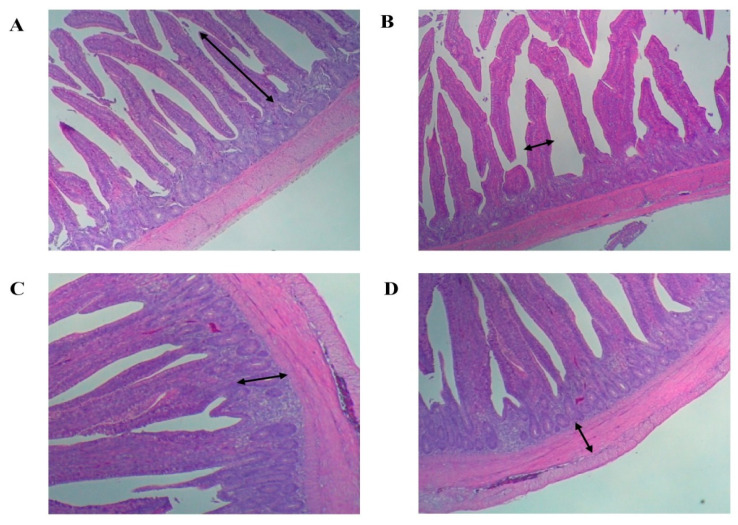
Intestine morphometric measurements (**A**). Villus height, (**B**). Villus width, (**C**). Crypt depth, (**D**). Mucosal depth. Bar: 100 µm.

Nutrient digestibility was not influenced by seaweed supplementation, as observed on d 14 and 28. However, the digestibility coefficients of dry matter (DM; *p* = 0.037) exhibited a linear improvement in broilers fed seaweed-supplemented diets on d 42, but no significant effects were observed for GE digestibility (Table 5). Dietary supplementation with seaweed led to a linear increase in *Lactobacillus* sp. (*p* = 0.035) counts, whereas *E. coli* (*p* = 0.027) counts were decreased on d 42 (Table 6). To contrast, no significant effects on *Salmonella* sp. counts were observed throughout the entire experiment (Table 6). Dietary supplementation with seaweed led to linear decreases in NH_3_ and H_2_S emissions on d 42. Conversely, no effects on total mercaptan, acetic acid, propionate, or butyrate were observed over the entire experiment (Table 7). The morphometric measurements of the chicken intestines can be found in Table 8, with these measurements represented in Figure 1. The dietary inclusion of seaweed influenced the morphology of the mucosa in the small intestine. Considering the jejunum, the average villus height and width were significantly enhanced in broilers fed seaweed-supplemented diets compared to the control broilers. 

The crypt depth and villus:crypt ratio appeared to be markedly influenced by seaweed supplementation, but the differences in values among treatments were not statistically significant (Table 8). The WHC (*p* = 0.0511) and cooking loss (*p* = 0.0129) decreased linearly with increasing levels of seaweed supplementation. A linear decrease in drip loss also was observed on d 7 (*p* = 0.0318). Additionally, the relative weights of the breast muscle (*p* = 0.033), bursa of Fabricius (*p* = 0.049), and gizzard (*p* = 0.018) increased linearly with increasing levels of seaweed supplementation (Table 9).

## 4. Discussion

Seaweeds or macroalgae are a good source of polysaccharides, which include complex carbohydrates that cannot be hydrolyzed in the upper gastrointestinal tract. Seaweeds, thus, are considered a healthy source of dietary fiber [24]. The polysaccharides derived from seaweeds also are considered potential prebiotics and cannot be digested in the small intestine, but they can survive bacterial fermentation in the large intestine and, hence, affect the intestinal microbiota [14]. Abudabos et al. [25] reported that dietary supplementation with seaweed in broilers resulted in decreased microbial abundance in the digestive tract and enhanced immune status, thereby improving the meat quality of the broilers. During the current study, dietary supplementation with red seaweed improved the growth performance of broilers in a linear manner, consistent with the findings of Wang et al. [26], who reported that broilers fed a seaweed-supplemented diet exhibited improvements in body weight. Similarly, Choi et al. [27] reported significant improvements in the BWG and feed intake of broilers upon supplementation with fermented seaweed; however, the feed efficiency was not affected.

Regarding terms of feed development in broiler production, the focus has expanded to include potential supplementation with natural products. Based on our results, the inclusion of seaweed in a corn/soybean meal-based diet led to significant increases in weight gain and feed efficiency, similar to the findings of Shi et al. [28]. Evans and Critchley [9] also showed that the dietary inclusion of seaweed enhanced bird health and productivity and improved the gut microflora. These benefits may be due to the immune-enhancing effect of red seaweeds. Concerning terms of their functional properties, polysaccharides such as fucans and alginic acid by-products in seaweeds exhibit anticoagulant, anti-inflammatory, antiviral, and antitumor activities [29]. Accordingly, dietary supplementation with seaweed has beneficial effects on broilers [30]. Our results also showed that the broilers fed red seaweed-supplemented diets exhibited improved nutrient digestibility, especially of DM. However, Nhlane et al. [31] obtained opposing results, with the nutrient digestibility in broilers apparently unaffected by dietary supplementation with seaweed. To date, only a few studies have been conducted on whether the dietary inclusion of seaweed improves nutrient digestibility in broilers. Improved nutrient digestibility is associated with better absorption of feed nutrients in birds as the feed passes through the digestive tract. Despite the conflicting results, we found that the villus height in the small intestine of supplemented birds was significantly taller, which is correlated with more efficient nutrient absorption and improved growth performance. Overall, we believe that dietary supplementation with seaweeds can improve the growth performance of, and nutrient digestibility in, broiler chickens by strengthening the intestinal integrity and immune system.

One of the goals of the present study was to determine whether dietary supplementation with seaweed would improve the blood profile of broiler chickens, as assessed based on levels of albumin, creatinine, uric acid, and WBC. Kang et al. [32] found that animals fed a dietary seaweed extract exhibited decreased oxidative stress, as shown by concentrations of glutamic oxaloacetic transaminase and glutamic pyruvic transaminase. To contrast, Lokaewmanee et al. [33] reported that supplementation with fermented brown seaweed had no significant effects on the blood profile of broilers. Research on the effects of seaweed supplementation on poultry blood profiles is limited, so we could not perform a comparative analysis. The conflicting results from previous experiments could have been due to variation in the seaweed dosage and differences in the composition of the additive of interest and diets. White and Venkatesh [34] determined that total cholesterol and triglycerides play major roles in humans, as they participate in animal cell membrane formation, fat transfer, and steroid hormone synthesis, and are correlated with blood sugar in the kidney.

During a recent study, Balasubramanian [35] demonstrated that gut health is a major factor regulating bird growth performance and, hence, affects the economics of poultry production. It also was noted that the intestinal microflora profile plays an important role in gut health. During the present study, dietary supplementation with red seaweed increased the *Lactobacillus* sp. count and decreased the *E. coli* count in the host gut. This finding agreed with the result of Bai et al. [36], who found decreased *E. coli* and increased *Lactobacillus* sp. counts in broiler chickens fed seaweed-supplemented diets. Earlier reports from Wang et al. [37] and Leonard et al. [38] indicated that *Laminaria japonica* powder could act as a potential medicine that enhances host resistance against gastrointestinal tract pathogens in weaner pigs by strengthening their antibody immune function. The powder also helped with controlling gastrointestinal function and promoting pig growth. Cecropins can destroy gram-positive and gram-negative bacteria in the intestinal tract by permeabilizing bacterial membranes [39]. Based on our results, we assume a similar beneficial effect from seaweed on gut microflora in poultry. Upon seaweed supplementation, seaweed components usually adhere to intestinal mucus and epithelial cells, and this attachment is believed to be key to their immunomodulatory effects [15].

Numerous studies have explored dietary manipulation strategies to lessen the environmental hazards from emissions of noxious gases such as NH_3_, H_2_S, and methyl mercaptan [40]. According to Yan and Kim [41], fecal noxious gas emission is associated with nutrient digestibility, with decreased digestibility resulting in reductions in the amount of microbial fermentation substrates in the large intestine and fecal noxious gas emission. Considering the present research, dietary supplementation with red seaweed significantly decreased NH_3_ and H_2_S concentrations but did not have significant effects on the concentrations of total mercaptan, acetic acid, propionate, and butyrate in fecal gas emissions. Notably, Wang et al. [42] stated that NH_3_, H_2_S, and methyl mercaptan affect the air quality and their emissions are a major problem in livestock production.

According to Offer and Knight [43], the most significant meat quality characteristic is the WHC. During the present study, the WHC tended to decrease with the level of seaweed supplementation, with a significant linear decrease with seaweed supplementation observed for drip loss on d 7, and linear associations were found for the relative weights of the breast muscle, bursa of Fabricius, and gizzard. Hossain et al. [44] reported that meat pH is commonly considered a direct indicator of the consistency of the muscle acid content, whereas drip loss is an indicator of meat quality [45]. Choi et al. [27] also found that organ weight was significantly influenced by fermented seaweed supplementation. To our knowledge, this is the first study on the meat quality of broilers fed diets supplemented with the seaweed *P. palmata.* Our results showed that the dietary inclusion of *P. palmata* had beneficial effects on the meat quality of broiler chickens, in terms of the WHC, drip loss, and organ weights. Nevertheless, antinutritional factors also may contribute to an increase in organ weights. It appears that seaweed supplementation might have improved the FCR and gut health (based on increases in villus height and width), thus resulting in a greater BWG compared to broilers fed the control diet. However, additional research is needed to verify our results.

## 5. Conclusions 

To summarize, the current study presents results from the first investigation of the effects of including marine red seaweed (*P. palmata*) in the diet of broilers, providing a foundation for further research. Given the positive effects on broiler growth performance, nutrient digestibility, fecal microbial counts, fecal gas emission, blood profile, histomorphology, and meat quality, dietary supplementation with *P. palmata* in broilers may be a suitable approach for promoting growth efficiency in the poultry industry.

## Figures and Tables

**Table 1:** Ingredient composition of experimental diets (as-fed basis).

Ingredients, g/kg	Starter (0–18 days)	Finisher (19–42 days)
Corn	547.7	629.1
Soybean meal, CP 48%	288.0	220.0
Corn gluten	95.0	85.0
Oil	23.0	25.0
Calcium carbonate	10.3	10.3
Dicalcium phosphate	20.0	17.6
Methionine, 99%	2.5	2.1
Lysine, 78.4%	4.5	4.7
Threonine, 98.5%	1.6	0.8
Salt	2.5	1.6
NaHCO_3_	1.7	1.0
Vitamin premix ^†^	1.0	1.0
Mineral premix ^‡^	1.0	1.0
Choline, 50%	1.2	0.8
Calculated composition		
Metabolizable energy (MJ/kg)	12.56	12.98
Analyzed composition, %		
Crude protein	22.99	20.51
Ether extract ^§^	3.840	3.680
Calcium	0.960	0.889
Available phosphorus	0.480	0.434
Digestible lysine	1.307	1.155
Digestible methionine	0.645	0.570
Digestible met-cys	0.984	0.878

^†^ Provided per kg of complete diet: 11,025 IU vitamin A; 1103 IU vitamin D_3_; 44 IU vitamin E; 4.4 mg vitamin K; 8.3 mg riboflavin; 50 mg niacin; 4 mg thiamine; 29 mg d-pantothenic; 166 mg choline; 33 μg vitamin B_12_. ^‡^ Provided per kg of complete diet: 12 mg Cu (as CuSO_4_∙5H_2_O); 85 mg Zn (as ZnSO_4_); 8 mg Mn (as MnO_2_); 0.28 mg I (as KI); 0.15 mg Se (as Na_2_SeO_3_∙5H_2_O). ^§^ Ether extract represents total fat content in the diet.

**Table 2:** The chemical compositions of seaweed (*H. palmata*).

Analysed Items	Contents
Moisture (%)	10.44
Crude protein (%)	18.48
Crude lipid (%)	1.69
Crude fiber (%)	1.83
Crude ash (%)	19.04
Carbohydrate (%)	50.35
Ca (%)	0.12
P (%)	0.33
Energy Kcal/100g	290.53
Total polyphenol (mg/kg)	2407.04
Total flavonoid (mg/kg)	211.14
Anti-trypsin factor (mg/kg)	0.37
Tannin (%)	4.90
Nitrate (mg/kg)	123.31
Oxalic acid (mg/kg)	694.31
Fe (%)	0.03
Zn (mg/kg)	72.74
Mg (%)	0.26
Vitamin A (IU/kg)	1102.06
Vitamin D_3_ (IU/kg)	70,214.59
Vitamin E (mg/kg)	9.48
Vitamin K_3_ (mg/kg)	16.55
Choline (mg/kg)	896.42

**Table 3:** The effect of red seaweed supplementation on growth performance in broilers ^1^.

Items	CON	Seaweed, %				SEM	*p*-Value	
		0.05	0.10	0.15	0.20		Linear	Quadratic
d 1 to 7								
BWG, g	108	115	110	112	114	3	0.2333	0.7537
FI, g	128	136	129	133	135	4	0.5087	0.9155
FCR	1.188	1.185	1.182	1.187	1.179	0.039	0.8936	0.9904
d 7 to 14								
BWG, g	247	259	252	261	256	9	0.5090	0.6510
FI, g	329	330	331	342	330	10	0.6648	0.6288
FCR	1.336	1.293	1.321	1.324	1.307	0.068	0.8950	0.9120
d 14 to 28								
BWG, g	691	689	704	717	719	15	0.0879	0.8951
FI, g	1204	1204	1228	1248	1247	19	0.0369	0.9106
FCR	1.748	1.752	1.746	1.747	1.740	0.039	0.8636	0.9129
d 28 to 42								
BWG, g	938	952	946	970	984	19	0.0829	0.7012
FI, g	1716	1739	1699	1700	1716	24	0.5976	0.7556
FCR	1.836	1.830	1.798	1.757	1.748	0.034	0.0280	0.9068
Overall								
BWG, g	1984	2015	2012	2060	2074	25	0.0075	0.8760
FI, g	3377	3409	3387	3423	3427	36	0.3234	0.9941
FCR	1.704	1.693	1.685	1.663	1.653	0.020	0.0499	0.8847

^1^ Abbreviation: CON (Basal diet, no antibiotic or additive); BWG, Body-weight gain; FI, Feed Intake; FCR, Feed conversion ratio; SEM, Standard error of means.

**Table 4:** The effect of red seaweed supplementation on blood profiles in broilers ^1^.

Items	CON	Seaweed, %				SEM	*p*-Value	
		0.05	0.10	0.15	0.20		Linear	Quadratic
Week 4								
Protein, g/dL	2.9	3.1	2.8	2.9	2.9	0.0	0.6974	0.6880
Albumin, g/dL	1.5	1.5	1.4	1.5	1.5	0.1	0.7466	0.2460
Globulin, g/dL	1.4	1.6	1.4	1.4	1.5	0.1	0.8727	0.9640
Creatinine, mg/dL	0.18	0.17	0.19	0.16	0.17	0.02	0.7000	0.9800
Uric acid, mg/dL	4.8	4.0	4.6	4.6	4.6	0.6	0.8933	0.6511
Triglyceride, mg/dL	74	72	74	72	74	13	0.9962	0.9579
Phosphorus, mg/dL	6.9	6.6	6.7	7.1	6.8	0.3	0.7317	0.7460
WBC, 10^3^/μL	23.64	24.75	24.69	23.90	26.79	2.20	0.4446	0.7363
RBC, 10^6^/μL	2.21	2.21	2.10	2.09	2.16	0.23	0.7749	0.7711
Lymphocyte, %	91.9	89.5	89.1	89.7	89.4	3.8	0.6971	0.7108
Finish								
Protein, g/dL	3.6	3.6	3.3	3.4	3.6	0.2	0.5504	0.6529
Albumin, g/dL	1.5	1.6	1.6	1.7	1.8	0.1	0.0029	0.4694
Globulin, g/dL	2.1	2.1	1.8	1.9	1.9	0.2	0.2919	0.9484
Creatinine, mg/dL	0.26	0.23	0.21	0.20	0.17	0.02	0.0068	0.8214
Uric acid, mg/dL	5.0	4.5	4.2	4.0	3.8	0.4	0.0469	0.6802
Triglyceride, mg/dL	36	34	37	36	32	7	0.9850	0.7045
Phosphorus, mg/dL	7.7	7.3	7.0	7.3	7.2	0.3	0.5539	0.7328
WBC, 10^3^/μL	23.64	24.75	25.19	25.40	26.79	0.98	0.0388	0.9301
RBC, 10^6^/μL	2.9	2.5	2.7	2.6	2.5	0.1	0.0675	0.4836
Lymphocyte, %	92.7	93.3	92.6	92.4	92.2	2.0	0.7786	0.7680

^1^ Abbreviations: CON (Basal diet, no antibiotic or additive); WBC, White blood cell count; RBC, Red blood cell count; SEM, Standard error of means.

**Table 5:** The effect of red seaweed supplementation on nutrient digestibility in broilers ^1^.

Items	CON	Seaweed, %				SEM	*p*-Value	
		0.05	0.10	0.15	0.20		Linear	Quadratic
d14								
Dry matter	73.68	73.71	73.86	73.29	72.85	0.78	0.4010	0.5738
Energy	73.50	73.50	74.55	73.59	73.54	0.64	0.9313	0.3812
d28								
Dry matter	73.45	73.02	73.43	73.83	73.00	0.62	0.9682	0.7295
Energy	73.73	72.74	73.60	74.10	73.34	0.73	0.8012	0.9698
d42								
Dry matter	69.97	71.19	71.40	71.77	72.29	0.76	0.0370	0.6646
Energy	70.82	71.11	71.29	71.46	71.26	0.71	0.5884	0.7161

^1^ Abbreviation: CON (Basal diet, no antibiotic or additive); SEM, Standard error of means.

**Table 6:** The effect of red seaweed supplementation on fecal microbial in broilers ^1^.

Items, log_10_cfu/g	CON	Seaweed, %				SEM	*p*-Value	
		0.05	0.10	0.15	0.20		Linear	Quadratic
d 14								
*Lactobacillus* sp.	7.60	7.64	7.62	7.62	7.67	0.07	0.6616	0.9091
*E. coli*	6.19	6.12	6.15	6.13	6.16	0.03	0.6520	0.1770
*Salmonella* sp.	2.45	2.35	2.32	2.40	2.38	0.06	0.6762	0.2183
d 28								
*Lactobacillus* sp.	7.67	7.65	7.69	7.71	7.70	0.08	0.6423	0.9639
*E. coli*	6.08	6.03	5.95	5.98	6.00	0.06	0.2769	0.2534
*Salmonella* sp.	2.19	2.14	2.08	2.10	2.15	0.09	0.7068	0.3945
d 42								
*Lactobacillus* sp.	7.76	7.77	7.83	7.91	7.94	0.07	0.0359	0.8060
*E. coli*	6.04	5.99	5.95	5.92	5.87	0.05	0.0272	0.9306
*Salmonella* sp.	2.18	2.17	2.12	2.06	2.11	0.09	0.3881	0.7599

^1^ Abbreviation: CON (Basal diet, no antibiotic or additive); SEM, Standard error of means.

**Table 7:** The effect of red seaweed supplementation on gas emission in broilers ^1^.

Items	CON	Seaweed, %				SEM	*p*-Value	
		0.05	0.10	0.15	0.20		Linear	Quadratic
d 14								
NH_3_, ppm	12.8	13.0	12.6	12.7	12.4	0.3	0.2510	0.6400
H_2_S, ppm	2.0	1.7	1.8	1.5	1.7	0.4	0.5794	0.7895
Total mercaptan, ppm	21.6	21.5	20.8	18.4	21.3	1.9	0.5387	0.5659
Acetic acid, ppm	1.6	1.5	1.3	1.5	1.3	0.4	0.4048	0.8126
Propionate, %	11.2	11.3	11.0	11.3	10.6	0.9	0.6864	0.7966
Butyrate, %	10.4	10.8	10.5	11.0	10.9	1.1	0.7483	0.9596
d 28								
NH_3_, ppm	12.5	12.6	12.0	10.8	14.1	1.0	0.6421	0.1253
H_2_S, ppm	1.9	2.4	2.1	2.5	1.9	0.3	0.9402	0.2762
Total mercaptan, ppm	24.7	27.1	25.9	23.7	25.3	2.3	0.7636	0.7499
Acetic acid, ppm	1.5	1.8	1.1	1.0	1.8	0.3	0.7770	0.1833
Propionate, %	14.7	12.8	15.3	15.0	12.9	1.8	0.8153	0.6458
Butyrate, %	13.9	12.0	12.1	13.2	15.4	1.6	0.4097	0.1225
d 42								
NH_3_, ppm	13.6	12.9	11.6	10.8	10.1	0.9	0.0051	0.9149
H_2_S, ppm	3.0	2.6	2.0	1.8	1.7	0.4	0.0153	0.5418
Total mercaptan, ppm	25.6	24.7	20.9	21.6	23.7	2.8	0.4549	0.3235
Acetic acid, ppm	1.8	1.7	1.5	1.7	1.2	0.3	0.2421	0.6842
Propionate, %	13.4	15.1	14.0	12.5	13.4	2.1	0.6981	0.8085
Butyrate, %	14.2	12.1	16.4	13.1	14.2	2.1	0.8907	0.9036

^1^ Abbreviation: CON (Basal diet, no antibiotic or additive); SEM, Standard error of means.

**Table 8:** The effect of dietary red seaweed supplementation on small intestinal morphology in broilers ^1^.

Items (µm)	CON	Seaweed, %				SEM	*p*-Value	
		0.05	0.10	0.15	0.20		Linear	Quadratic
Villus height	434.9	448.28	510.78	476.32	518.27	24.51	0.049	0.451
Villus width	80.55	77.16	101.12	126.93	110.03	7.36	0.001	0.368
Crypt depth	87.71	112.33	115.53	87.04	122.77	6.96	0.513	0.718
Villus:Crypt ratio	5.18	4.07	4.53	5.56	4.24	0.33	0.360	0.758
Mucosal depth	117.53	81.99	88.37	80.1	85.45	9.50	0.036	0.069

^1^ Abbreviation: CON (Basal diet, no antibiotic or additive); SEM, Standard error of means.

**Table 9:** The effect of red seaweed supplementation on meat quality in broilers ^1^.

Items	CON	Seaweed, %				SEM	*p*-Value	
		0.05	0.10	0.15	0.20		Linear	Quadratic
pH value	7.56	7.76	7.76	7.80	7.72	0.09	0.2003	0.1303
Breast muscle color								
Lightness (L*)	56.87	55.41	54.79	55.18	56.00	0.88	0.4846	0.1043
Redness (a*)	10.93	10.69	11.68	11.77	10.50	0.68	0.9193	0.2533
Yellowness (b*)	15.05	14.93	15.99	15.94	16.02	0.63	0.1483	0.7602
Water Holding capacity, %	49.26	46.15	49.43	40.99	41.19	3.28	0.0511	0.6803
Cooking loss	22.95	22.10	21.23	20.11	19.98	0.93	0.0129	0.7353
Drip loss, %								
d 1	1.11	1.26	1.87	1.84	1.47	0.34	0.2412	0.2076
d 3	3.12	2.43	3.28	3.70	2.51	0.53	0.9725	0.4833
d 5	2.92	3.32	4.53	3.87	3.69	0.57	0.2533	0.1667
d 7	7.48	7.13	7.05	6.79	6.04	0.45	0.0318	0.5667
Relative organ weight, %								
Breast muscle	18.25	18.54	18.90	19.38	20.89	0.86	0.0331	0.4347
Liver	3.28	3.25	3.22	3.21	3.14	0.21	0.6448	0.9383
Bursa of Fabricius	0.09	0.11	0.11	0.12	0.14	0.02	0.0492	0.9233
Abdominal fat	2.95	2.41	2.14	2.07	2.84	0.42	0.6658	0.0838
Spleen	0.21	0.21	0.21	0.21	0.21	0.02	0.7964	0.9842
Gizzard	1.50	1.54	1.67	1.70	1.77	0.09	0.0189	0.9189

^1^ Abbreviation: CON (Basal diet, no antibiotic or additive); SEM, Standard error of means.

## Data Availability

The data presented in this study are available on request from the corresponding author.
